# Hepatoprotective effect of *Stachys pilifera* ethanol extract in carbon tetrachloride-induce hepatotoxicity in rats

**DOI:** 10.1080/13880209.2017.1302484

**Published:** 2017-03-19

**Authors:** Esmaeel Panahi Kokhdan, Kyomarth Ahmadi, Heibatollah Sadeghi, Hossein Sadeghi, Fahemeh Dadgary, Nazanin Danaei, Mahmoud Reza Aghamaali

**Affiliations:** a Department of Biology, Faculty of Science, University of Guilan, Rasht, Iran;; b Biochemistry Department, Faculty of Medicine, AJA University of Medical Sciences, Tehran, Iran;; c Medicinal Plants Research Center, Yasuj University of Medical Sciences, Yasuj, Iran;; d Cellular and Molecular Research Center, Yasuj University of Medical Sciences, Yasuj, Iran;; e School of Nursing, AJA University of Medical Sciences, Tehran, Iran

**Keywords:** AST, ALP, hepatotoxicity, MDA

## Abstract

**Context:***Stachys pilifera* Benth (Lamiaceae) has long been used to treat infectious diseases, respiratory and rheumatoid disorders in Iranian folk medicine. Antitumor and antioxidant activity of the plant have been reported.

**Objective:** The study was designed to assess the hepatoprotective activity of ethanol extract of *Stachys pilifera* in carbon tetrachloride (CCl_4_)-induced hepatotoxicity in rats.

**Materials and methods:** The rats were randomly divided into six equal groups (*n* = 7). Group I was treated with normal saline; Group II received CCl_4_ (1 mL/kg. i.p., twice a week) for 60 consecutive days; Groups III, IV and V were given CCl_4_ plus *Stachys pilifera* (100, 200 and 400 mg/kg/d,p.o.); Group VI received the extract (400 mg/kg/d, p.o.). Histopathological analysis and measurement of serum aspartate aminotransferase (AST), alanine aminotransferase (ALT), alkaline phosphatase (ALP), malondialdehyde (MDA), total protein (TP) and albumin (ALB) were performed.

**Results:** CCl_4_ caused a significant increase in the serum levels of AST, ALT, ALP and MDA as well as decreased ALB, and TP serum levels (*p* < 0.001). The extract (200 and 400 mg/kg/d) significantly normalized the CCl_4_-elevated levels of ALT, AST, ALP and MDA (*p* < 0.001). The extract (200 and 400 mg/kg/d) also increased the serum levels of TP compared to CCl_4_ group (*p*< 0.01). The extract (200 and 400 mg/kg/d) also decreased the histological injuries (inflammation and fatty degeneration) by CCl_4_.

**Discussion:** The results revealed that the *Stachys pilifera* extract could provide considerable protection against CCl_4_ hepatotoxicity in rats that may be related to its antioxidant properties.

## Introduction

The liver is the main organ involved in metabolic functions. Hepatic damage is associated with alteration of these metabolic functions. Liver dysfunction induces by habitual repeated alcohol consumption, exposure to some xenobiotics, and/or drug interactions. Management of the liver disorders remains a controversial subject (Kumar et al. 2011). In absence of a reliable and effective agent for prevention and treatment of liver diseases, many researchers are focusing on introducing hepatoprotective compounds from natural products (Levy et al. 2004). Therefore, medicinal plants have usually been an effective and good option for prevention or treatment of the liver dysfunction (Valiathan 1998).

*Stachys pilifera* Benth (Lamiaceae) grows in tropical and subtropical countries. This genus is represented in Iran by 34 species that 13 of them are endemic (Zargari 1995). Presence of flavonoids, phenylethanoid glycosides, diterpenes, saponins, terpenoids, and steroids has been reported in the phytochemical evaluation of *Stachys* species (Garjani et al. 2004; Javidnia et al. 2006; Biglar et al. 2014). Several biological studies have shown consideration anti-inflammatory, antioxidant, antibacterial and anti-hepatitis effects of this genus (Maleki et al. 2001; Khanavi et al. 2005; Sonboli et al. 2005; Hajhashemi et al. 2007; Ebrahimabadi et al. 2010). *Stachys pilifera* is one of the endemic species in Iran that aerial parts of the plant are used in Iranian conventional medicine for the treatment of different diseases such as asthma, rheumatoid arthritis, and infections (Zargari 1995). Antioxidant, antitumor, and antimicrobial effects of the *n*-butanol extract of *Stachys pilifera* have been reported in previous studies (Farjam et al. 2011; Sadeghi et al. 2014). In view of this, the present study was designed to investigate the effect of the *Stachys pilifera* ethanol extract in carbon tetrachloride (CCl_4_)-induced hepatotoxicity in rats.

## Materials and methods

### Plant material

Aerial parts of *Stachys pilifera* including stems and leaves were collected from Kakan in Yasuj, Iran at the end of spring of 2015. The plant was authenticated by Dr. A. Jafari from Department of Botany, Center for Research in Natural Resource and Animal Husbandry, Yasuj University, Yasuj, Iran, where a voucher specimen (herbarium No. 1897) was deposited. The plant leaves were dried far from direct light, and then powdered. The powder was kept in a closed container in 4 °C.

### Extract preparation

Dried leaf powder plant (500 g) was extracted three times with 1500 mL mixture of EtOH-H_2_O (70:30) at 400 °C by maceration for four days. After this time, the extract were filtered, the solution was evaporated by rotary evaporator in 40 °C. The rest was kept at 4 °C prior to being tested (Pourmorad et al. 2006; Mehraban et al. 2014; Sadeghi et al. 2014).

### Chemicals

Trichloroacetic acid (TCA), tiobarbituric acid (TBA), (CCl_4_) diethyl ether and other solvents were obtained from Merck, Germany. The assay kits for the determination of ALT, AST, ALP, ALB and TP were purchased from Pars Azemun, Tehran, Iran.

### Animals

Forty-two adult male Wistar rats weighing 180–220 g were purchased from Razi Institute in Shiraz, Iran. The animals were maintained in controlled temperature (23 ± 2˚C) and a 12 h dark/light cycle and were allowed to take standard laboratory feed and tap water. The animals were kept fasted 2 h before and 2 h after drug administration.

The animals were randomly assigned to six groups, each consisting of 7 rats as follows:

Group I: the animals received intraperitoneal injection (i.p.) of olive oil (1 mL/kg, twice a week) and normal saline (0.5 mL/d, p.o.) for 60 consecutive days.

Group II: the animals received i.p. injection of CCl_4_ as a 50% solution in olive oil (1 mL/kg twice a week) for 60 consecutive days and 0.5 ml normal saline (0.5 mL/d, p.o.) for 60 consecutive days.

Group III: the animals received i.p. injection of CCl_4_ as a 50% solution in olive oil (1 mL/kg, twice a week) and the *Stachys pilifera* extract (100 mg/kg/d, p.o.) for 60 consecutive days.

Group IV: the animals received i.p. injection of CCl_4_ as a 50% solution in olive oil (1 mL/kg, twice a week) and the *Stachys pilifera* extract (200 mg/kg/d, p.o.) for 60 consecutive days.

Group V: the animals received i.p. injection of CCl_4_ as a 50% solution in olive oil (1 mL/kg, twice a week) and the *Stachys pilifera* extract (400 mg/kg/d, p.o) for 60 consecutive days.

Group VI: the animals only received the *Stachys pilifera* extract (400 mg/kg/d, p.o.) for 60 consecutive days.

The doses of *Stachys pilifera* were selected according our previous study (Sadeghi et al. 2014).

### Measurement of serum biochemical parameters

The blood samples were collected and centrifuged at 2500 rpm, for 15 min. The obtained serum was analyzed for measurement of activities of aspartate aminotransferase (AST), alanine aminotransferase (ALT), alkaline phosphatase (ALP), and the levels of total protein (TP) and albumin (ALB). The assays were performed by a colorimetric method using commercially available kits (Pars Azemun, Iran) (Aghel et al. 2007; Mohan et al. 2007; Sharma & Vasudeva 2011; Sadeghi et al. 2016).

### Determination of lipid peroxidation

The serum level of MDA was performed according to the according to our previous study (Buege & Aust 1978; Akbartabar Toori et al. 2015; Sadeghi et al. 2016). According to this method, 375 mg of TBA was dissolved in 2 mL of chlorhydric acid (HCl, 0.25 N), followed by 15 g of trichloroacetic acid (TCA) for a total volume of 100 mL. The solution was heated in a water bath at 50˚C till TBA properly dissolved. Then, 0.5 mL of serum was mixed with 2 mL of TCA-TBA-HCl. Next, the solution was heated for 15 min in a boiling water bath. After cooling, the flocculent precipitate was removed by centrifugation (2500 *g*, 15 min). Finally the absorption of supernatant was determined at 535 nm against a blank that contained all reagents except the serum sample. Serum MDA concentration was expressed as nmol/mL (Sharma et al. 2006).

### Histopathology

After blood collection, the rats were sacrificed and their livers were removed and fixed in 10% buffered formaldehyde solution for 1 week. Then, the paraffin sections were prepared (Automatic tissue processor, Autotechnique) and cut into 3–4 mm slices using a rotary microtom. The slices were then stained with Hematoxylin-Eosin dye and studied for histopathological changes (Akbartabar Toori et al. 2015).

### Statistical analysis

All data were expressed as mean ± SD, and analyzed by one-way analysis of variance (ANOVA) (SPSS 21 for windows) followed by Dunnett and Tukey post-test. *p*-Value <0.05 was considered to show significant differences for all the comparisons.

## Results

### Biochemical estimation

As presented in Table 1, intoxication with CCl_4_ caused a significant increase in the serum levels of ALT, AST, and ALP compared to the saline group (*p* < 0.001). Pretreated with the ethanol extract of *Stachys pilifera*, at the doses of 200 and 400 mg/kg/d, significantly decreased the CCl_4_-elevated serum levels of ALT, AST and ALP (*p* < 0.001). *Stachys pilifera* at the dose of 100 mg/kg/d also considerably reduced the serum levels of ALT, and ALP (*p* < 0.01). The serum levels of TP and ALB was considerably reduced due to intoxication with CCl_4_ (*p* < 0.01). *Stachys pilifera* extract, at doses of 200 and 400 mg/kg/d markedly normalized the serum levels of TP and ALB compared to CCl_4_ group (*p* < 0.001), while 100 mg/kg/d of the extract was observed to non-significantly reduced the elevated levels of the indicated parameters. Analysis of the serum levels of TP and ALB and activities of ALT, AST, ALP confirmed no significant differences in the biochemical parameters between the control group and the animals treated with the extract at a dose of 400 mg/kg/d (*p* > 0.05).

**Table 1.** Effect of *of Stachys pilifera* ethanol extract on liver function tests in CCl_4_-induced liver toxicity in rats.

Treatment group	ALT (U/l)	AST (U/l)	ALP (U/l)	TP (g/dL)	ALB (g/dL)
Control	77 ± 6	147 ± 11	308 ± 33	6.9 ± 0.31	3.8 ± 0.13
CCl_4_	167 ± 14***	222 ± 15***	800 ± 54***	6.23 ± 0.081***	2.7 ± 0.31***
100 S.P + CCl_4_	122 ± 14***###	222 ± 6***	695 ± 63***##	6.65 ± 0.15##	2.85 ± 0.22*
200 S.P + CCl_4_	97 ± 8*###	193 ± 14.6***###	519 ± 34***###	6.98 ± 0.14###	2.95 ± 0.18
400 S.P + CCl_4_	79 ± 3###	160 ± 8###	382 ± 20**###	7.1 ± 0.25###	2.76 ± 0.37
400 S.P only	70 ± 10###	157 ± 8###	362 ± 39###	7.08 ± 0.14###	4.01 ± 0.25

Values are presented as Mean ± S.D. S.P: ethanolic extract of *Stachy spilifera*; ALT: alanine aminotransferase; ALP: alkaline phosphatase; AST: aspartate aminotransferase; ALB: albumin; TP: total protein.

* *p* < 0.05.

** *p* < 0.01.

*** *p* < 0.001 vs. control group.

## *p* < 0.01.

### *p* < 0.001 vs. CCl_4_ group, *n* = 7.

### Determination of lipid peroxidation

**Figure 1.**
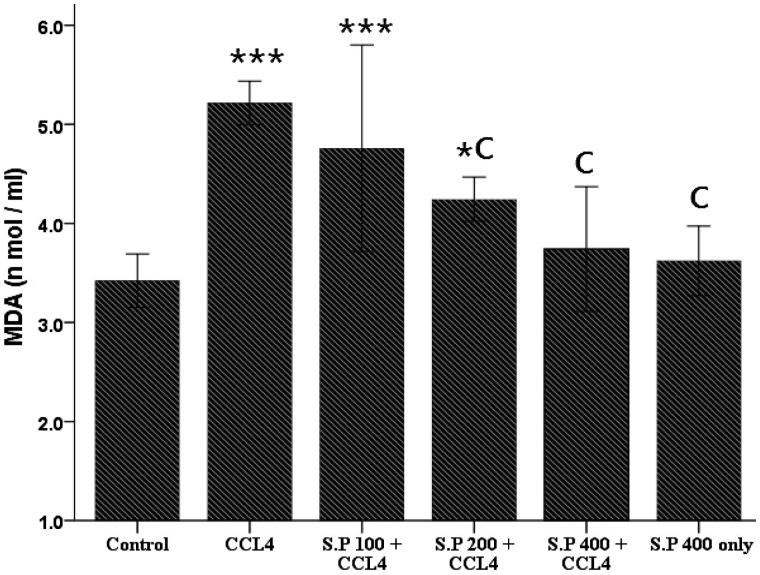
Effect *of Stachys pilifera* ethanol extract on the serum MDA level in CCl_4_-induced hepatotoxicity in rats. Data are expressed as Mean ± S.D. *p < 0.05 and ***p < 0.001 vs. control group. C, *p* < 0.001 vs. CCl_4_ group, *n* = 7.

As shown in Figure 1 the serum concentration of MDA were significantly enhanced following injection of CCl_4_ compared to the saline group (*p* < 0.001). According to the results, the extract at doses of 200 and 400 mg/kg/d, considerably reduced the MDA levels in the rats that received CCl_4_ (*p* < 0.001). There was no difference in the MDA levels in the rats treated with *Stachys pilifera* (400 mg/kg/d) compared to the saline group (*p* > 0.05).

### Histopathological studies

**Figure 2.**
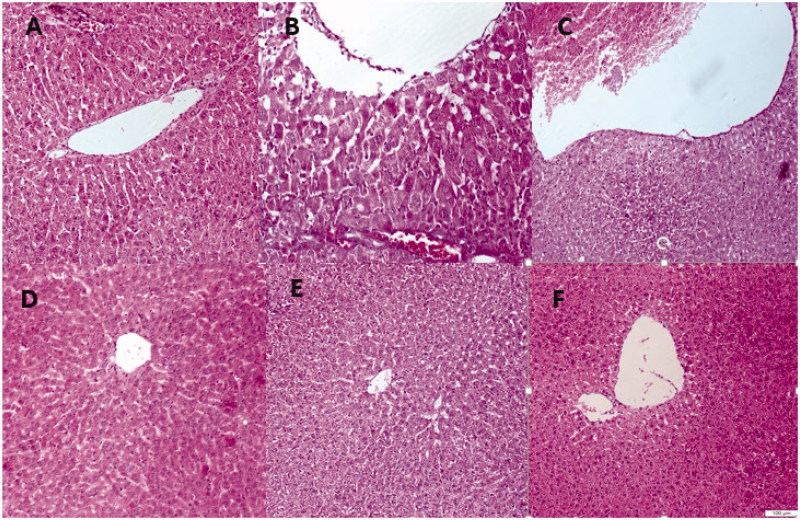
H&E staining of liver tissues isolated from CCl_4_-exposed rats demonstrated that CCl_4_ poisoning resulted in excessive formation of fatty acid, necrosis of Kupffer cells around the central vein and netrophil infilteration (B). The ethanol extract at the doses of 200 and 400 mg/kg/d reversed the liver injures by CCl_4_, towards normal pattern (C and D). The extract at the dose of 100 mg/kg/d did not show a considerable protective effect on the pathological changes induced by CCl_4_ (E). Examination of the liver sections from the rat treated only with 400 mg/kg/d *Stachys pilifera* showed normal architecture (F) similar to control group (A).

As illustrated in Figure 2, histology of the liver section from the saline group showed normal hepatic cells each with well-preserved cytoplasm, prominent nucleus, and central vein (Figure 2(A)). Intoxication of the animals with CCl_4_ caused the normal architecture of liver to be entirely lost. CCl_4_ poisoning resulted in excessive formation of fatty acid, necrosis of Kupffer cells around the central vein and netrophil infilteration (Figure 2(B)). The ethanol extract at the doses of 200 and 400 mg/kg/d reversed the liver injury by CCl_4_, towards normal pattern (Figure 2(C,D)). However, the extract at the dose of 100 mg/kg/d did not show a considerable protective effect on the pathological changes induced by CCl_4_ (Figure 2(E)). Examination of the liver sections from the rat treated only with 400 mg/kg/d *Stachys pilifera* showed normal architecture similar to saline group.

## Discussion

The results of the present study confirmed the hepatoprotective effect of *Stachys pilifera* in CCl_4_-induced liver toxicity. CCl_4_ has been widely used to induce hepatic injuries in the animal models of liver disease (Lee et al. 2007; Rudnicki et al. 2007; Desai et al. 2012). CCl_4_ generates an experimental damage that histologically looks like viral hepatitis. Hepatotoxicity commences with the change in endoplasmic reticulum, which results in the release of metabolic enzymes located in the intracellular structures.

One of the ways for estimating of the extent of hepatic damage is through the determination of the serum level of cytoplasmic enzymes such as ALT; AST and ALP leak from damaged liver cells into the blood, which is indicates the centrilobular necrosis, ballooning degeneration and cellular infiltration (Ramaiah 2007). In this study, CCl_4_ intoxication considerably increased the serum levels of ALT, AST and ALP in the animals, a marker of cellular leakage and failure in activities of cell membrane in liver (Yang et al. 2011). Reduction of the serum concentration of TP by CCl_4_ is a further indicator of liver toxicity. CCl_4_ disrupts and dissociates polyribosomes on endoplasmic reticulum, which lead to decreasing protein synthesis (Kumar et al. 2009). The *Stachys pilifera* extract (200 and 400 mg/kg/d) reversed the elevated levels of ALT, AST and ALP induced by CCl_4_ toxicity. In this regard, we have shown that the extract significantly increased the abnormal plasma levels of TP and ALB. The findings are consistent with a previous study of that showed methanol extracts of four *Stachys* species seeds reduced the levels of ALT, AST and ALP in paracetamol or CCl_4_-induced hepatotoxicity (Kukic-Markovic et al. 2011).

It has been reported that lipid peroxidation, reducing activity of antioxidant enzymes and generation of free radicals are the primary reasons of CCl_4_-induced hepatic injury (Srivastava & Shivanandappa 2006). Free radicals/reactive oxygen species (ROS) and oxidative stress play a central role in liver toxicity of CCl_4_ (Loguercio & Federico 2003; Vitaglione et al. 2005; Tang et al. 2010). The cleavage of CCl_4_ leads to the formation of highly unstable free radicals (CCl_3_ or CCl_3_O_2_), to initiate lipid peroxidation (Recknagel et al. 1989). MDA is a secondary product of poly-unsaturated fatty acids peroxidation (Amat et al. 2010) and serves as a main marker to estimate the levels of lipid peroxidation (Cho et al. 2013). Furthermore, the level of lipid peroxidation is an indicator of cell membrane damage (Kepekçi et al. 2013). In the present study, *Stachys pilifera* ethanol extract (200 and 400 mg/kg/d) considerably normalized the abnormal elevating serum levels of MDA in the CCl_4_-induced hepatotoxic rats.

Histopathological examination of liver biopsies confirmed our biochemical findings. Injection of CCl_4_ induced a variety of hepatic histological changes including congestion in central vein, destroyed lobular structure and leukocyte infiltration. These changes significantly inhibited by 200 and 400 mg/kg/d *Stachys pilifera.*

According to the results the exact underlying mechanisms for this protective effect of *Stachys pilifera* in the model of CCl_4_-induced liver injury is not clear, but it is important to note that the plant is rich in phenolic compounds (Sadeghi et al. 2014). These compounds exhibit a variety of biological and pharmacological activities, including anti-inflammatory, antioxidant and antibacterial activities (Sadeghi et al. 2014). Furthermore, our results confirmed that *Stachys pilifera* ethanol extract reduced the elevated levels of MDA. Therefore, it is possible the *Stachys pilifera* extract exerted its protective effects through the antioxidant effect or scavenging free radicals.

## Conclusions

In conclusion, the results of the present study clearly demonstrated hepatoprotective effects of the *Stachys pilifera* ethanol extract in CCl_4_-induced hepatic damage in rats. These protective effects may be, at least in part, related to antioxidant properties of the extract.
